# Modifying a covarying protein–DNA interaction changes substrate preference of a site-specific endonuclease

**DOI:** 10.1093/nar/gkz866

**Published:** 2019-10-11

**Authors:** Marc Laforet, Thomas A McMurrough, Michael Vu, Christopher M Brown, Kun Zhang, Murray S Junop, Gregory B Gloor, David R Edgell

**Affiliations:** Department of Biochemistry, Schulich School of Medicine and Dentistry, Western University, London, ON N6A 5C1, Canada

## Abstract

Identifying and validating intermolecular covariation between proteins and their DNA-binding sites can provide insights into mechanisms that regulate selectivity and starting points for engineering new specificity. LAGLIDADG homing endonucleases (meganucleases) can be engineered to bind non-native target sites for gene-editing applications, but not all redesigns successfully reprogram specificity. To gain a global overview of residues that influence meganuclease specificity, we used information theory to identify protein–DNA covariation. Directed evolution experiments of one predicted pair, 227/+3, revealed variants with surprising shifts in I-OnuI substrate preference at the central 4 bases where cleavage occurs. Structural studies showed significant remodeling distant from the covarying position, including restructuring of an inter-hairpin loop, DNA distortions near the scissile phosphates, and new base-specific contacts. Our findings are consistent with a model whereby the functional impacts of covariation can be indirectly propagated to neighboring residues outside of direct contact range, allowing meganucleases to adapt to target site variation and indirectly expand the sequence space accessible for cleavage. We suggest that some engineered meganucleases may have unexpected cleavage profiles that were not rationally incorporated during the design process.

## INTRODUCTION

Mechanisms that regulate enzyme specificity may not be fully apparent from structural or biochemical studies. Molecular covariation analysis utilizes multiple sequence alignments to predict intramolecular amino acid co-dependencies with the hypothesis that covarying residues contribute to protein structure and function (1–3). Predicted residue pairs often lie within contact distance of each other thus providing a straightforward interpretation of covariation where direct contacts disrupted by substitution of one residue can be functionally compensated for by covariation in the other residue (4). However, some analyses identified covariation between non-contacting residue pairs that may indirectly impact activity through conformational dependencies (5). Covariation analysis has also been used to predict intermolecular dependencies between DNA-binding proteins and their targets to inform binding-site predictions for modular zinc finger and homeobox transcription factors (6–8). When applied to protein families with rapidly evolving DNA-binding interfaces (9,10), covariation predictions of intermolecular protein–DNA dependencies could reveal direct and indirect mechanisms that regulate DNA interactions to provide useful starting points for engineering new specificity.

**Figure 1.**
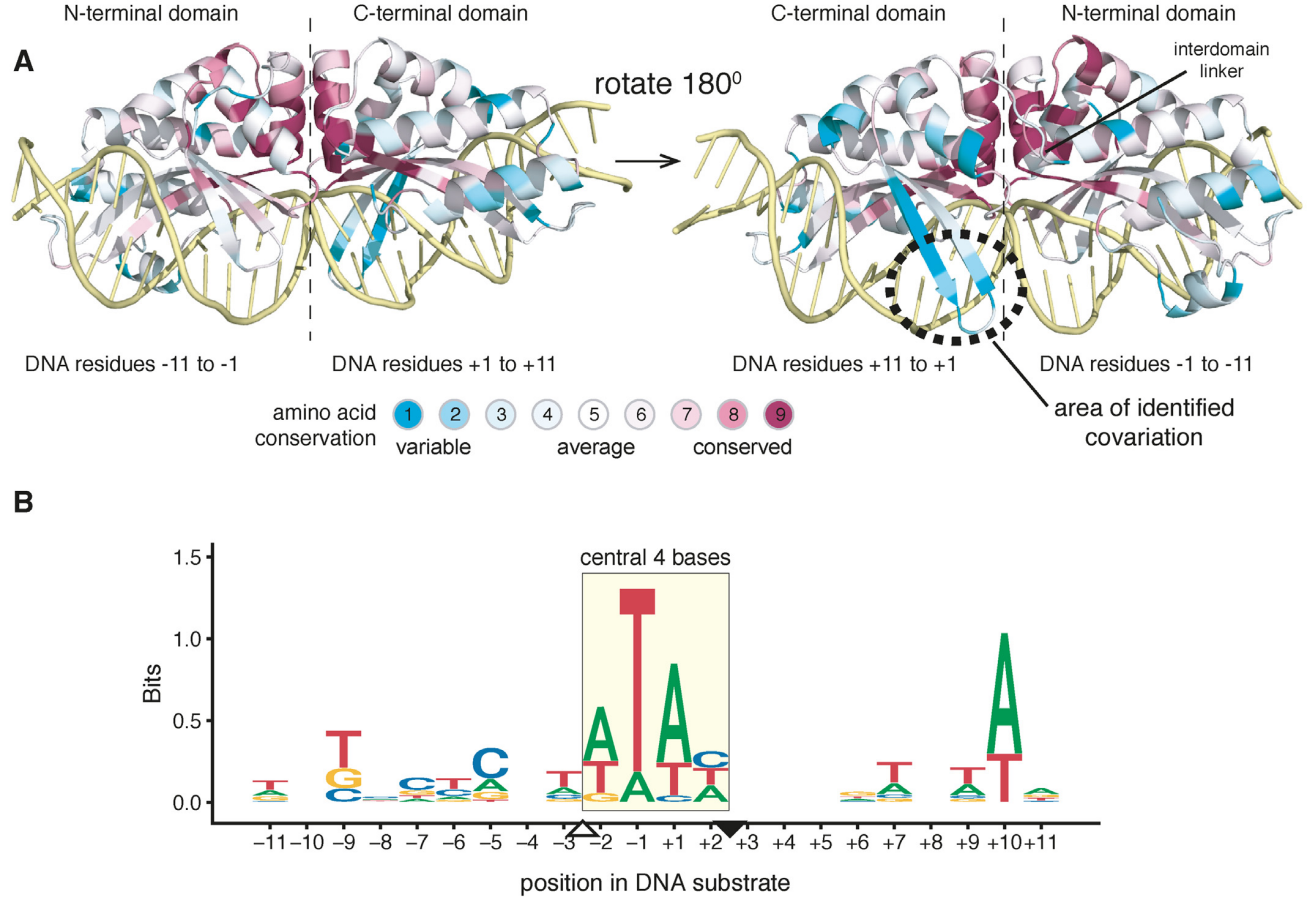
Overview of LAGLIDADG structure and evolutionary conservation. (**A**) Evolutionary conservation of LAGLIDADG homing endonucleases overlaid on the structure of the monomeric I-OnuI (modified from PDB ID: 3QQY). The N- and C-terminal domains of I-OnuI are indicated, as are the (−) and (+) halves of the DNA substrate. The color scheme is based on a conservation score generated by the ConSurf program, with variable residue positions given a score of 1 and highly conserved residue positions a score of 9 (55). (**B**) Logos alignment of aligned DNA target sites used in the MI analysis. The substrates are labeled according to standard nomenclature with the central 4 bases indicated by a rectangle and the top- and bottom-nicking sites indicated by downward and upward facing rectangles, respectively.

LAGLIDADG endonucleases (or meganucleases) are site-specific DNA endonucleases that belong to a class of mobile genetic elements termed homing endonucleases (11,12). Meganucleases are typically encoded within self-splicing genetic elements (introns and inteins) where they function as drivers of genetic invasion by cleaving target sites that lack the intron or intein (13). Meganucleases are identified by the conserved class-defining LAGLIDADG amino acid motif that forms the inter-subunit interface and catalytic center of the enzyme (14–16). Outside of the LAGLIDADG motifs, meganucleases exhibit low amino acid conservation (Figure 1A). Each meganuclease has a defined native DNA target site that can range from 14–22 bp in length. Target sites can be pseudo-palindromic for dimeric meganucleases, such as I-CreI (16), or asymmetric for single-chain monomeric versions, such as I-OnuI (17). Cleavage occurs in the center of the substrate at the so-called central 4 bases (Figure 1B). Meganucleases interact with their target sites in a sequence-tolerant manner, accommodating varying degrees of nucleotide variation without significant loss of activity (9,18–19). In general, greater nucleotide variation is accommodated in the flanking DNA rather than at positions closer to the central 4 bases.

One intriguing question regarding the biology of meganucleases relates to the evolution of new target specificity (10,20). As mobile genetic elements, meganucleases must balance evolutionary persistence within a population of existing target sites with the sampling of new target sites for transposition and invasion. Sequence-tolerant interactions with DNA substrates allows meganucleases to bind new or divergent sites distinct from their native site. From an evolutionary perspective, meganuclease variants with novel specificity profiles that permitted sampling of more divergent sites would have been successful in promoting transposition and colonization relative to variants with restricted specificity.

Insights into how meganucleases evolve new DNA-binding specificity have largely come from structural and biochemical studies of meganuclease–DNA interactions (reviewed in (21)). A combination of direct readout of flanking bases and indirect readout of shape and sequence of the central 4 bases determines meganuclease specificity. In particular, DNA-contacting residues in β-sheets that straddle the major grooves of substrate make direct (or indirect through water molecules) contacts to bases. DNA-contacting residues are sometimes found in the loops that connect the β-sheets, but in general the loops show little conservation in sequence or length among meganucleases. Thus, variation in base-contacting residues is one evolutionary mechanism for changing the targeting range of meganucleases. Engineering strategies that target these base-contacting residues to generate meganucleases for genome editing at non-native sites have largely recapitulated this evolutionary mechanism (22–24). Computational approaches that optimize protein structure around residue–base interactions have also shown promise (25–28). However, the complexity of meganuclease–DNA interactions, including both direct and indirect mechanisms to readout DNA shape and sequence, coupled with the fact that not all meganuclease redesigns on non-native targets are successful, strongly suggests that meganuclease binding and cleavage specificity are not fully understood. A more comprehensive understanding of mechanisms that regulate meganuclease specificity will provide insight into evolutionary pathways for innovation of specificity, and better inform meganuclease redesign studies for gene editing.

Here, we take advantage of the observation that meganucleases coevolve with their DNA target sites (9,29) and use (MI) analyses to identify residue–DNA covariation that contribute to meganuclease specificity. MI analyses of meganuclease–DNA interactions can potentially provide complementary information about specificity determinants to that obtained from structural or other computational approaches. In particular, MI analysis can identify residue–DNA dependencies in non-DNA contacting positions that indirectly influence meganuclease specificity. Using multiple sequence alignments of meganucleases and their cognate target sites, we identified and experimentally validated one residue–base combination that indirectly influences specificity of the I-OnuI meganuclease at the +2 position of the central 4 bases. Our findings show that substitutions in covarying residues can create new base-specific contacts as well as modulate cleavage preference, and suggest that some engineered meganucleases may have unanticipated cleavage preference that would only be revealed by extensive substrate profiling (30).

## MATERIALS AND METHODS

### Mutual information analyses

Twenty-eight monomeric meganucleases with known target sites were retrieved from the literature (31). Cn3D was used to produce a structure guided alignment of the meganucleases. Target sites were separately aligned using the top and bottom strand scissile phosphates, trimmed to 22 nts, and appended to the end of the corresponding meganuclease sequence in the alignment (Supplementary File S1). The MIp Toolset was used for MI calculations (32,33). Corrections to the MI calculation were applied to remove the average phylogenetic entropy resulting in a phyogentically corrected MI score (MIp) (34). MIp scores were converted to a *Z*-score (Zpx). DNA target sites were randomly shuffled using custom R scripts and the SeqinR package (35). MIp calculations were repeated 10 000 times with these randomly shuffled DNA target sequences. Output from the MIp calculations is provided in Supplementary Table S1.

### Library construction and directed evolution

A modified *Escherichia coli* two-plasmid selection was used to screen activity of I-OnuI and I-OnuI libraries on various target sites as previously described (36,37). This screen uses a toxic plasmid (pTox) that expresses a DNA gyrase toxin under control of a *lac* promoter into which DNA target sites are cloned (using AflIII and BglII restriction sites). I-OnuI (or libraries of I-OnuI) are expressed from a second compatible carbenicillin-resistant plasmid (pEndo) under control of the pBAD arabinose-inducible promoter. Survival in the assay is dependent on the endonuclease expressed from pEndo cleaving the target site on pTox, and is expressed as a ratio of colony counts under selective (expressing) versus non-selective (non-expressing) conditions. I-OnuI libraries were constructed by splicing-overlap extension polymerase chain reaction (PCR) using oligonucleotides with NNS (where N is any nucleotide and S is a G or C) in positions corresponding to codons to be randomized. All constructs were sequenced to confirm proper assembly. Plasmids (50 ng) of wild-type I-OnuI or I-OnuI libraries were transformed into NovaXGF’ (Novagen) cells harbouring pTox, recovered at 37°C for 10 min, and then transferred to tubes containing 1 ml of 2× YT induction medium (16 g/l tryptone, 10 g/l yeast extract and 5 g/l NaCl, 100 μg/ml carbenicillin, 0.02% L-arabinose) and incubated with shaking at 37° for 1 h. Cultures were then diluted and spread onto selective (2× YT plus 0.02% L-arabinose, 0.005 mM isopropyl β-D-1-thiogalactopyranoside (IPTG)) and non-selective media (2× YT plus 0.02% glucose) plates to obtain a survival ratio (37), or grown in liquid culture at 200 rpm for growth curves.

### *In vitro* barcoding assay

I-OnuI proteins were purified as previously described (37). The barcoding substrates were generated by the PCR to amplify fragments of 2200, 1800, 1600 and 1320 bp from pTox (38). Each fragment size corresponds to a different nt at the +3 target site position. The appearance of a product was used as an indicator of successful cleavage. A single pot cleavage reaction (5 nM substrate, 50 mM Tris–HCl (pH 8.0), 100 mM NaCl, 10 mM MgCl2, 1 mM dithiothreitol (DTT) and 250 nM protein) was incubated at 37°C and 10 μl aliquots were removed at various times, and stopped with stop solution (50 mM ethylenediaminetetraacetic acid (EDTA), bromophenol blue, 30% glycerol and 0.2% sodium dodecyl sulphate). Time points were resolved on a 0.8% agarose gel, imaged using an AlphaImager 3400 instrument and quantitated using spot densitometry. Three biological replicates were performed for each protein–substrate combination, and modeled with the lm function in R to give a cleavage rate for each substrate that was normalized to the A+3 substrate to yield a relative cleavage rate.

### Substrate depletion assays using deep sequencing

An I-OnuI substrate library randomized in positions +2 to +5 was created as previously described (39). This library (5 nM) was mixed with purified I-OnuI proteins at 1600 nM in reaction buffer (50 mM Tris–HCl pH 8.0, 100 mM NaCl, 10 mM MgCl_2_ and 1 mM DTT), and incubated at 37°C. Aliquots were taken at 0, 5, 10, 15, 30, 60 and 120 min and stopped with stop buffer. Five biological replicates were performed for each protein and substrate combination. The remaining supercoiled substrate was separated by gel electrophoresis, and extracted. The target site region was amplified by PCR with primers that incorporated barcodes and Illumina adaptor sequences. PCR products were pooled and sequenced on an Illumina Mi-Seq at the London Regional Genomics Centre at Western University. Custom Perl scripts were used to deconvolute the barcodes and extract target site regions from reads, allowing for one mismatch in the up- or down-stream region flanking the randomized region (note that CAAC is the wild-type sequence at positions +2 to +5). A count table for each of the 256 substrates was generated, and analyzed by the compositional analysis software ALDEx 2.0 (40). This analysis transforms the raw count data using a center log ratio transformation (log2) (41). For each substrate and protein combination, the log2 values of relative substrate abundance across all time points were used to calculate a rate of decay and a standard error of the estimate using the lm function in R (Supplementary Table S2). Data were plotted using the R statistical and graphics framework.

### Protein expression and purification

Wild-type and K227Y/D236A I-OnuI were cloned between the NcoI and NotI sites of plasmid pProExHta (Invitrogen and Life Technologies). The 6×-His tagged proteins were expressed in *E. coli* strain BL21(DE3) (New England Biolabs) at 16°C for 16 h by induction with 1 mM IPTG. Cells were harvested at 6000 × *g* for 15 min and resuspended (40 ml/1 g of cell pellet) in binding buffer containing 50 mM Tris–HCl, pH 8.0, 500 mM NaCl, 5 mM imidazole, and 10% (vol/vol) glycerol. Cells were lysed using an EmulsiFlex-C3 high-pressure homogenizer followed by sonication for 30 s. Cell lysates were clarified by centrifugation at 29 000 × *g* for 30 min at 4°C, and the supernatant loaded onto a nickel-charged 5 ml HiTrap column (GE Healthcare Life Sciences). The column was washed sequentially with 10 column volumes of binding buffer supplemented with 30 and 60 mM imidazole prior to elution with binding buffer containing 300 mM imidazole. Eluted protein was buffer exchanged into 50 mM Tris–HCl (pH 8.0), 150 mM NaCl, 5 mM dithiothreitol, 1 mM EDTA and 10% (vol/vol) glycerol prior to digestion with Tobacco Etch Virus (TEV) protease at a molar ratio of 1:25 TEV to I-OnuI. The protein mixture was buffer exchanged into binding buffer and run over an equilibrated 5-ml HiTrap column. The flow through, containing pure I-OnuI protein, was exchanged into storage buffer [50 mM Hepes⋅NaOH, pH 7.5, 150 mM NaCl, 20 mM MgCl_2_, and 5% (vol/vol) glycerol], concentrated to 10 mg/ml and stored at −80°C.

### Crystallization substrates and procedures

I-OnuI A+3 substrate: 5′-CTTTCCACTTATTCAACCTTTTACCC-3′; 5′-GGTAAAAGGTTGAATAAGTGGAAAGG-3′. A+3G substrate: 5′- CTTTCCACTTATTCGACCTTTTACCC-3′; 5′- GGTAAAAGGTCGAATAAGTGGAAAGG -3′. The A+3 substrate was used for crystallization with I-OnuI variant K227Y/D236A to generate the structure 6BD0, while the A+3G substrate was crystallized with both wild-type and K227Y/D236A I-OnuI to generate structures 6BDA and 6BDB, respectively. Protein preparations were combined with hybridized substrate duplexes in a 1:1.5 ratio (protein:substrate) and incubated at 4°C for 4 h to promote complex formation. Crystallization was performed using the hanging-drop vapor diffusion method by equilibrating equal volumes (1 μl) of protein/DNA complex and mother liquor over 0.8 ml of 1.2 M ammonium sulfate. Crystals of wild-type I-OnuI in complex with A+3G substrate were generated with mother liquor containing 0.2 M potassium formate and 20% (w/v) polyethylene glycol 3350. Crystals of I-OnuI variant K227Y/D236A in complex with A+3 substrate were generated with mother liquor containing 0.18 M tri-ammonium citrate and 20% (w/v) polyethylene glycol 3350. Crystals of I-OnuI variant K227Y/D236A in complex with A+3G substrate were generated with mother liquor containing 0.2 M di-ammonium phosphate and 20% (w/v) polyethylene glycol 3350. All crystals were grown at 20°C, and crystal growth was achieved within 7 days for all constructs.

### Structure determination

All crystals were directly flash frozen in liquid nitrogen without further cryoprotection. Diffraction data were collected using Beam 17-ID at the Advanced Photon Source of Argonne National Labs. Three hundred and sixty degrees of data were collected in quarter degree wedges for all crystals. Images were indexed and integrated using iMOSFLM (42). Reflections were scaled and merged using the Aimless and Ctruncate modules from CCP4i (43,44). Merged reflections were then used for molecular replacement in PHENIX (45). An existing I-OnuI structure (PDB ID: 3QQY) was used as a search model for molecular replacement. Models were refined using iterative cycles of manual rebuilding in COOT (46) and the refine module from Phenix until the Rfree and Rwork values converged and geometry statistics reached suitable ranges. Structural parameters for DNA substrate structures were analyzed using 3DNA (47). A summary of x-ray data collection and structure refinement statistics can be found in Supplementary Table S3.

### Accession numbers

Coordinates and structure factors have been deposited in the Protein Data Bank with accession numbers 6BDA, 6BD0, 6BDB.

## RESULTS

### Mutual information predictions of meganuclease–DNA target site covariation

**Figure 2.**
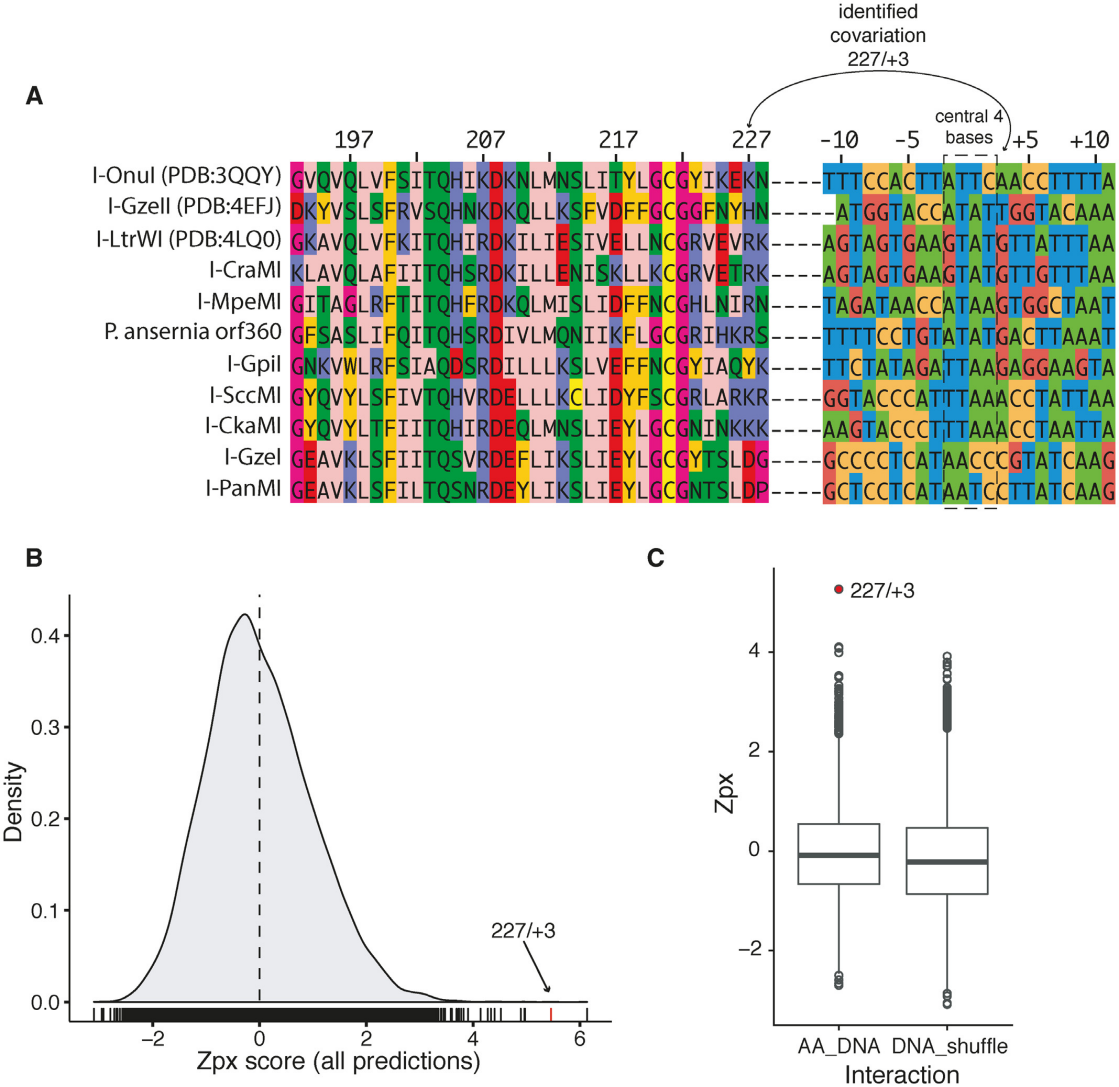
Evolutionary-normalized MI predictions of protein–DNA co-variation. (**A**) A portion of the multiple sequence alignment used in the MI analyses. Shown are aligned residues 193–228 of 11 characterized meganucleases with their mapped DNA substrates concatenated to the end of each sequence (the full alignment is provided in Supplementary File S1). The central 4 bases of each target site is indicated by a dashed rectangle. The residue pair 227/+3 identified by MI is indicated by solid line. Amino acid residues are colored by using the Zappo color scheme of JalView using physicochemical properties. DNA target sites are colored by nucleotide. All numbering is relative to I-OnuI (3QQY). (**B**) Density plot of Zpx scores from MI analyses of the LHE-DNA alignment. The dotted vertical line represents the mean Zpx value. The Zpx score for the 227/+3 pair is indicated by an arrow and red vertical line. (**C**) Boxplots of Zpx scores binned by amino acid-DNA contacts (AA_DNA) and scores for the 227/+3 pair from 10 000 MI analyses with randomly shuffled DNA sequences (DNA_shuffle).

To identify putative specificity-determining features of meganucleases that are distinct from those previously identified by structural studies, we used computational MI methods to predict protein–DNA covariation from multiple sequence alignments. We assembled a concatenated multiple-sequence alignment of 28 meganucleases with mapped 22-bp DNA cognate target sites to use in MI predictions (Figure 2A and Supplementary File S1) (31,48–49). Because mis-alignments in protein or DNA sequences lead to false-positive predictions (32), we constrained our analyses to meganucleases with structurally or biochemically characterized target sites rather than predicted target sites. Target sites were aligned based on the position of the two meganuclease cut sites that flank the central 4 bases, and trimmed to 9 bases on either side (Figure 1B). Phylogenetically corrected mutation information (MIp) scores were obtained for the meganuclease–DNA alignment (32–34), converted to *Z*-scores (Zpx), and plotted to determine the highest-scoring interactions (Figure 2B). All numbering is relative to the I-OnuI structure, PDB ID: 3QQY, and target site DNA sequence (Figure 2A) (17). The highest Zpx score corresponded to a previously validated intramolecular AA-AA pairing (A21-G177) (37) (Table 1 and Supplementary Table S1), indicating that concatenating the meganuclease alignment with DNA did not impact prediction of biologically validated co-variation predictions. The second highest Zpx score corresponded to an AA-DNA pair, K227 and A+3 (Figure 2B).

**Table 1.** High-scoring pairs from MIp calculations

Residue 1	Residue 2	Zpx
A21	G177	6.1
K227	A+3	5.5
N75	K274	5.0
C-9	C+2	5.0
G260	K262	4.9
T16	H93	4.5

Amino acid positions (single letter code) are numbered relative to the I-OnuI crystal structure (3QQY), while residues with + or − signs represent nucleotide positions in the I-OnuI substrate as shown in Figure 1. All predictions are available in Supplemental Table S1.

To test the sensitivity of the co-variation statistic to the protein–DNA alignment, we randomly shuffled the DNA target sequences and recalculated the Zpx score for the K227 and +3 DNA positions from MI analysis of 10 000 independent shufflings of the DNA sequence (Figure 2C). This analysis showed that the Zpx score for the K227/A+3 pair from the unshuffled alignment fell outside of the distribution of scores from the shuffled alignments. Based on these analyses, the 227/+3 pair was prioritized for further analysis because K227/+3 is localized within a module of meganuclease residues that contact DNA (17). Other high-scoring residue–DNA predictions, notably R7/-4 and L15/+3, were given lower priority because R7 and L15 are distant to the protein–DNA interface.

### Substitutions in the +3 position of the I-OnuI substrate impact activity

**Figure 3.**
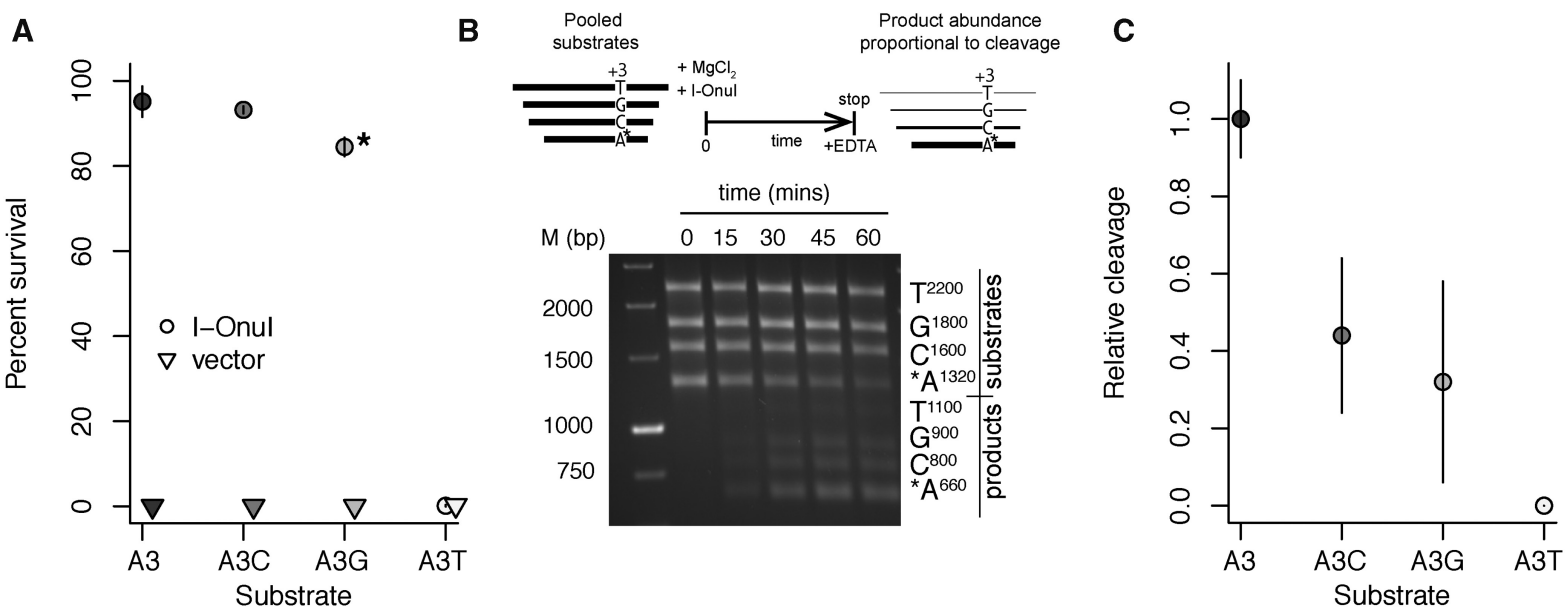
Nucleotide substitutions at position +3 impact I-OnuI *in vivo* and *in vitro* activity. (**A**) I-OnuI *in vivo* survival on +3 substrates tested in an *Escherichia coli* two-plasmid selection system. Circles represent mean survival values from at least three independent experiments with I-OnuI, and inverted triangles mean survival values from from at least three independent experiments with the vector. The lines represent the range of error. Symbols are shaded by substrate identity. The asterisk (*) indicates a slow-growth colony phenotype associated with the A+3G substrate. (**B**) Top, schematic of *in vitro* barcoding assay where substrates of different lengths contain nucleotide substitutions in the +3 position. Incubation over time generates products of different lengths. Bottom, representative cleavage assay analyzed by gel electrophoresis in 0.8% agarose with lengths of +3 substrates and products indicated. (**C**) Relative cleavage of the +3 substrates derived from quantitative analysis of product and substrate ratios from panel (B). Circles indicate the mean relative cleavage from at least 3 independent experiments and the lines represent the range of error.

We used an *E. coli* two-plasmid selection system to assess the relevance of the K227/+3 co-variation prediction by determining the impact of nucleotide substitutions at the +3 position (37,39,50). This assay reports on endonuclease cleavage of a target site cloned into a plasmid expressing the *ccdB* DNA gyrase toxin under inducible control. Activity (percent survival) is reported as a ratio of colony counts under inducing versus non-inducing conditions. We first tested survival of wild-type I-OnuI on substrates with nucleotide substitutions in the +3 position, revealing a preference in the order A∼G>C>T (Figure 3A). Next, we purified the wild-type I-OnuI protein for use in *in vitro* barcode cleavage assays (38), where cleavage is simultaneously monitored on four different length substrates, three of which have substitutions in the +3 position. The I-OnuI cleavage site is positioned in the middle such that cleavage will generate a single product of defined length for each substrate (Figure 3B). Cleavage rates for each substrate relative to the wild-type substrate are determined from time-course assays. As shown in Figure 3C, the relative cleavage rates of the I-OnuI wild-type protein agree with the *in vivo* data. Collectively, these data indicate that the substrate preference is in the order A>G∼C>T. Our *in vitro* analysis agrees well with previous *in vitro* profiling of single nucleotide substitutions across the I-OnuI DNA substrate (17).

### I-OnuI variants in positions K227 and D236 rescue activity on DNA substrates with substitutions in the +3 position

**Figure 4.**
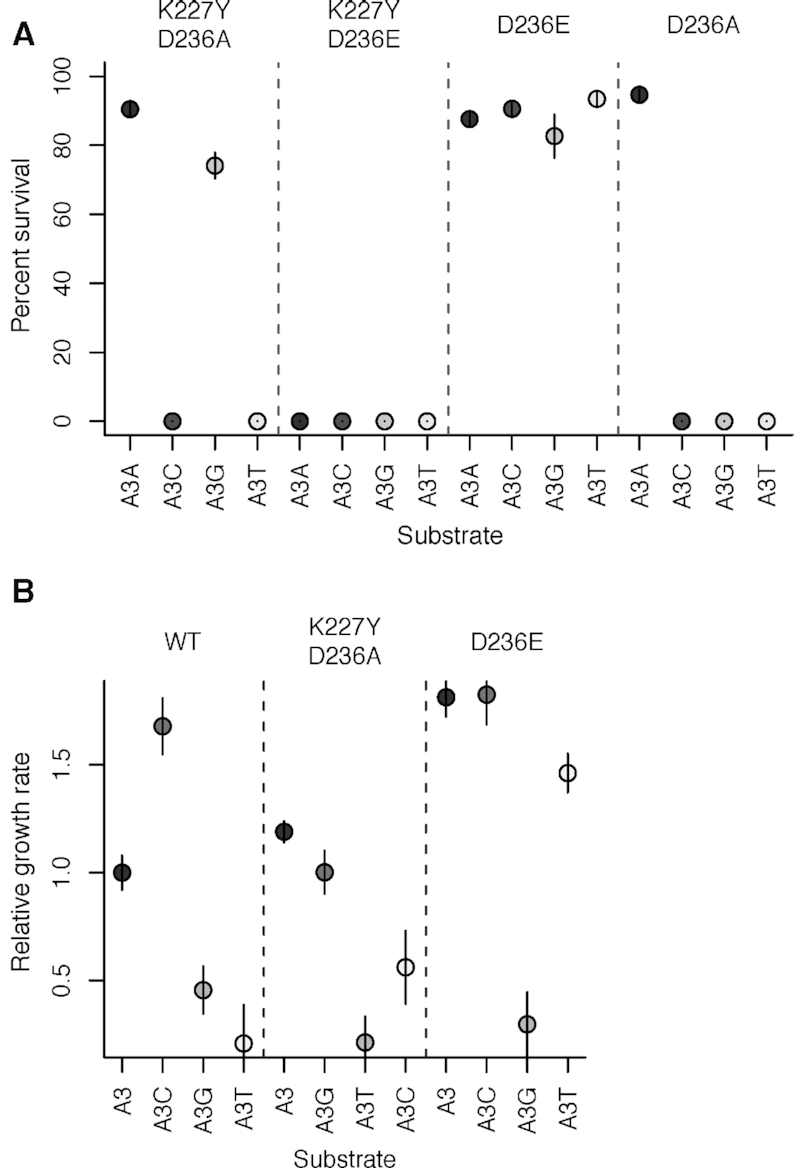
I-OnuI variants rescue activity on DNA substrates with +3 substitutions. (**A**) Plot of I-OnuI variant *in vivo* survival on +3 substrates tested in an *E. coli* two-plasmid selection system. (**B**) Plot of *in vivo* growth rate of I-OnuI wild-type and variant proteins in liquid media. Growth is relative to the wild-type protein on the A+3 substrate. Circles indicate the mean relative cleavage from at least three independent experiments and the lines represent the range of error.

We generated a library of I-OnuI variants with all possible amino acid substitutions in position 227 (the 1NNS Library). This randomized library was constructed to test the hypothesis that amino acid changes in the covarying position 227 should compensate for nucleotide substitutions in the +3 position in DNA substrate. The library was screened in directed evolution experiments using the two-plasmid selection system to identify I-OnuI variants with activity on the A+3T and A+3G substrates. Multiple experiments revealed negligible growth on the A+3T and A+3G substrates, whereas robust growth was observed on the wild-type A+3 substrate (Table 2). Further examination of the published I-OnuI structure near position +3 revealed a network of water-mediated contacts between K227, W234 and D236, and the DNA substrate. All three positions (K227, W234, D236) were subsequently randomized in a single library to select for active variants (the 3NNS library). We repeatedly isolated a K227Y/D236A variant that survived on the A+3G substrate, and a D236E variant that survived on the A+3T substrate (Table 2). No variants with substitutions in position W234 were isolated. Deconvolution of the most common variants revealed that the K227Y/D236A duo preferred A+3 and A+3G substrates, whereas the K227Y/D236E double mutant was inactive on all substrates. The D236E variant survived on all +3 substrates but exhibited a small colony phenotype on the A+3G substrate (Figure 4A). The D236A variant survived only on the A+3 wild-type substrate. We also determined the relative growth rates in liquid culture for active I-OnuI variants (Figure 4B). The growth rates agreed well with the solid media survival data. In particular, the D236E variant had a slow relative growth rate on the A+3G substrate, agreeing with the small colony phenotype and possibly explaining why it was not isolated in directed evolution screens with the 3NNS library and the A+3G substrate.

**Table 2.** Isolation of I-OnuI variants on substrates with substitutions in A+3

Library	Substrate	Substitutions	Independent isolations
1NNS	A+3	K227K	2
		K227S	2
	A+3G	none	none
	A+3T	none	none
3NNS	A+3	K227G/W234/D236S	2
		K227G/W234/D236V	2
		K227R/W234/D236	1
	A+3G	K227Y/W234/D236A	5
	A+3T	K227/W234/D236E	15

The 1NNS library refers to position 227, while 3NNS library refers to positions 227, 234 and 236. All positions are reported for the 3NNS library regardless of whether a substitution was found at each position.

### Substrate profiling reveals changes in nucleotide preference at position +2

**Figure 5.**
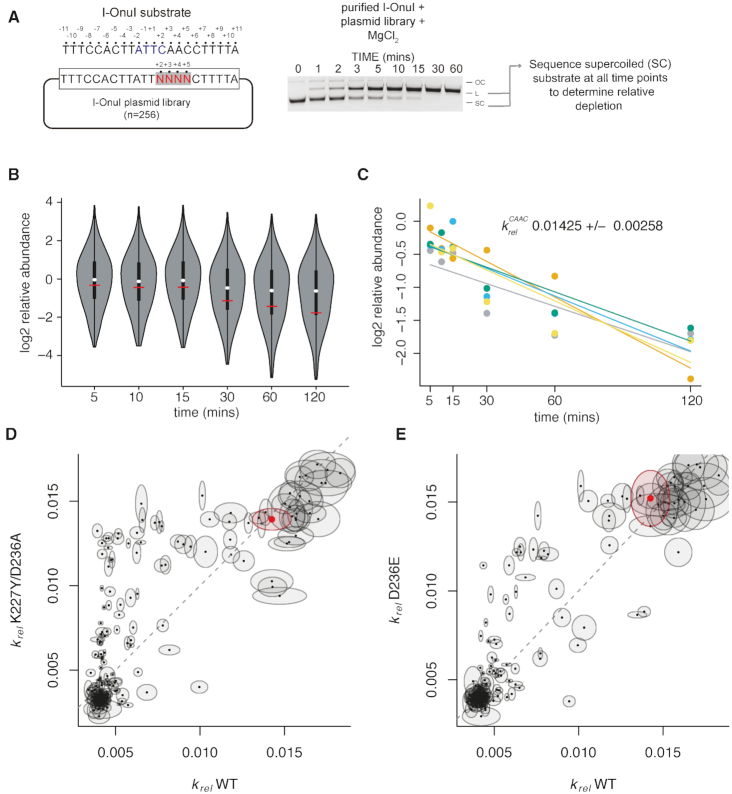
Profiling of cleavage specificity using *in vitro* depletion and deep sequencing. (**A**) At left is a schematic of the *in vitro* plasmid depletion assay with a I-OnuI substrate randomized in nucleotide positions +2,+3,+4,+5. A representative image of a time-course cleavage assay is shown on the right. OC, open circular plasmid; SC, supercoiled plasmid; L, linear plasmid. (**B**) Violin plot of relative log2 substrate depletion over time for all 256 substrates. At each time point, the white dot represents the median of the data, the black rectangle the interquartile range and the thin vertical black lines the distribution of data outside of the interquartile range. The horizontal red line represents the relative abundance of the wild-type substrate (CAAC) in positions +2,+3,+4,+5. (**C**) Example of *k_rel_* estimate for I-OnuI wild-type protein and the CAAC wild-type DNA substrate. Each colored point represents a log2 relative abundance value calculated from independent experiments with lines representing the best fit. *k_rel_* for each substrate and protein combination are provided in Supplementary Table S2. (**D** and **E**) Plots of *k_rel_* values highlighting differences between I-OnuI WT and K227Y/D236A, and I-OnuI WT and D236E. Each point represents a *k_rel_* value for an individual substrate, with the ellipses representing the standard deviation estimated for the WT and variant enzymes. The red dot and ellipse indicates the *k_rel_* of the wild-type substrate (CAAC).

**Figure 6.**
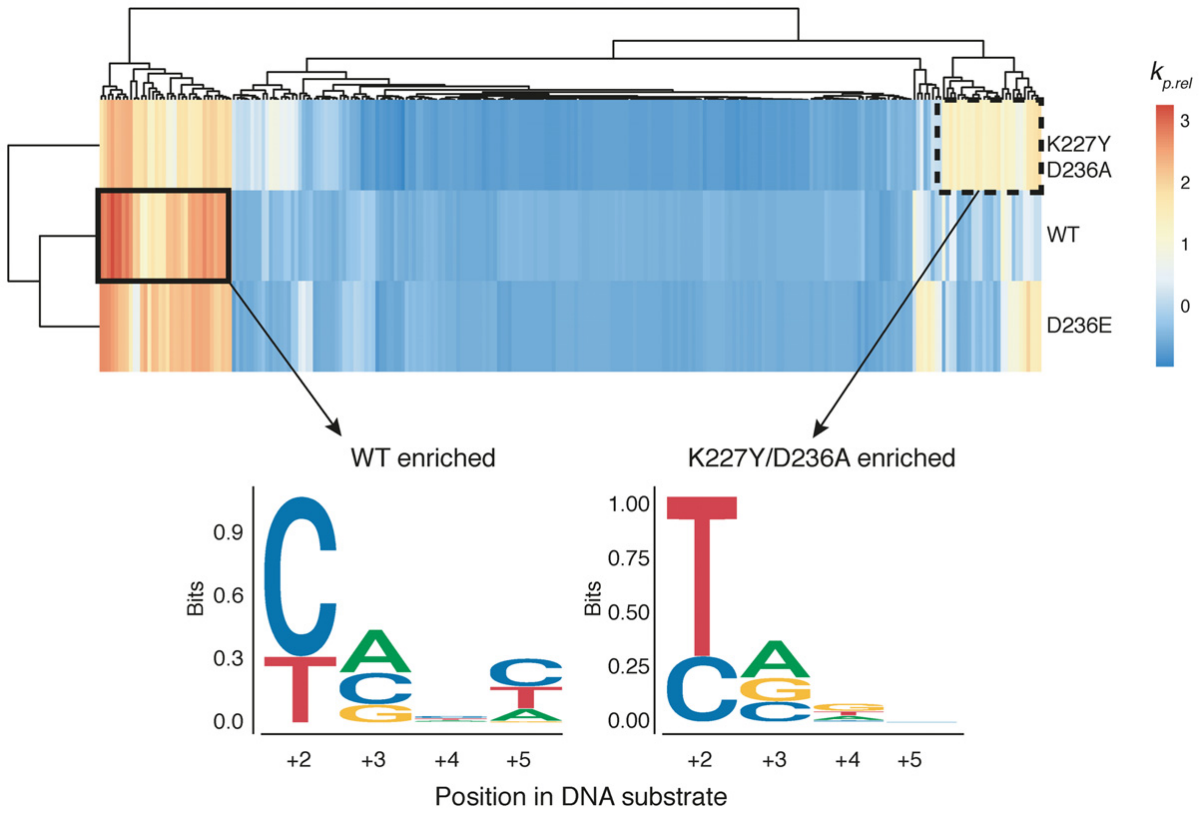
Heatmap of substrates enriched by I-OnuI WT, K227Y/D236A and D236E with logos of nucleotide preference for the WT and K227Y/D236A proteins indicated below. The sequence logos were generated from sequences preferentially enriched by each variant relative to other variants as indicated by the solid rectangle (for the wild-type enzyme) and the dashed rectangle (for the K227Y/D236A enzyme). The wild-type sequence at these positions is CAAC.

Profiling of I-OnuI activity at individual positions does not take into account the impact of nucleotide identity or DNA structure at adjacent positions. We created a supercoiled plasmid substrate library where the I-OnuI substrate was randomized at positions +2, +3, +4 and +5, representing 256 possible variants (for reference, the wild-type substrate is CAAC) (Figure 5A). The supercoiled plasmid library was incubated with purified I-OnuI, I-OnuI K227Y/D236A or I-OnuI D236E, aliquots taken at intervals over a 120-min time course, and the I-OnuI target site was amplified by PCR from undigested plasmid with bar-coded primers for Illumina sequencing. We performed at least five experimental replicates for each enzyme, allowing us to determine a relative substrate depletion rate (*k_rel_*) for each enzyme on each of the 256 possible substrates (Figure 5B and C and Supplementary Table S2). Plotting *k*_*rel*_ values for the K227Y/D236A and D236E variants relative to the wild-type I-OnuI protein revealed changes for a subset of substrates (Figure 5D and E; Supplementary Table S2). Notably, the *k*_*rel*_ for the wild-type CAAC substrate was unchanged for each variant. To determine trends in substrate preference, we plotted the *k*_*rel*_ values as a heatmap (Figure 6). The most significant finding from this analysis was a switched T to C preference at position +2 for the K227Y/D236A variant relative to the wild-type and D236E enzymes.

### Remodeling of a hairpin loop and DNA backbone shift explains substrate preference of the D227Y/D236A variant

**Figure 7.**
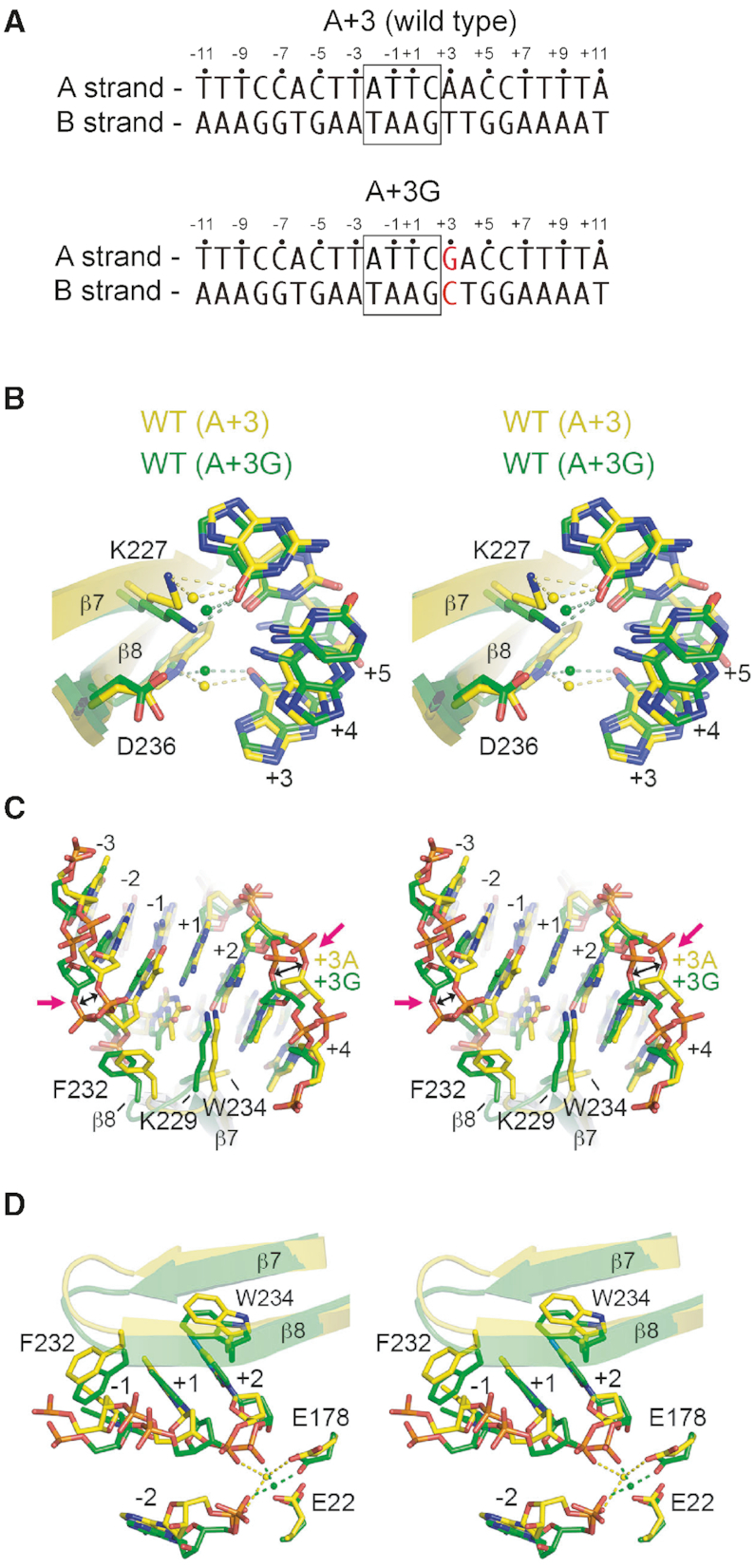
Structures of wild-type I-OnuI with cognate A+3 and A+3G substrates. In all panels, the I-OnuI protein is colored according to substrate, with A+3 as yellow and A+3G as green. Note that the I-OnuI/A+G3 complex is post-cleavage and both DNA strands are cut. (**A**) Schematic of substrates used in crystallization experiment. (**B**) The network of direct and water-mediated contacts between K227, W234, D236 and bases +3, +4 and +5. (**C**) Distortions in the phosphate backbone of the A+3G substrate relative to the cognate substrate. (**D**) Perturbations of the active site residues E178 and E22 of the A+3G substrate relative to the cognate substrate. All panels are stereo images.

We first crystallized the I-OnuI wild-type protein with A+3G substrate (PDB ID: 6BDA) and compared it to the A+3 bound structure (PDB ID: 3QQY) to gain insight into why the A+3G substrate was poorly cleaved by I-OnuI (Figure 7A and Supplementary Table S3). The I-OnuI/A+3G co-crystal was solved in the post-cleavage state with both DNA strands cut. In both structures, K227, W234 and D236 (located on adjacent antiparallel-strands 7 and 8) orchestrate a network of direct and water-mediated contacts that stabilize interaction with nucleotides at positions +3, +4 and +5 within the major groove (Figure 7B). Since this network remains largely intact in the A+3G co-crystal, only minor structural reorganization is required for β-strands 7/8 and the connecting loop to accommodate the A+3G substitution. Notably however, substitution of A to G at the +3 position causes a local distortion in the sugar–phosphate backbone at position +3 and also results in an ∼1.5Å outward translation of W234 away from the G+3 base (Figure 7C). This movement is further propagated within the hairpin ends of strands 7/8 leading to base and backbone shift at positions +1, −1, −2 and −3. Ultimately, the coordination and geometry of the active site is perturbed (Figure 7D) such that the rate of cleavage on the A+3G substrate is approximately 3-times slower than on the wild-type A+3 substrate.

**Figure 8.**
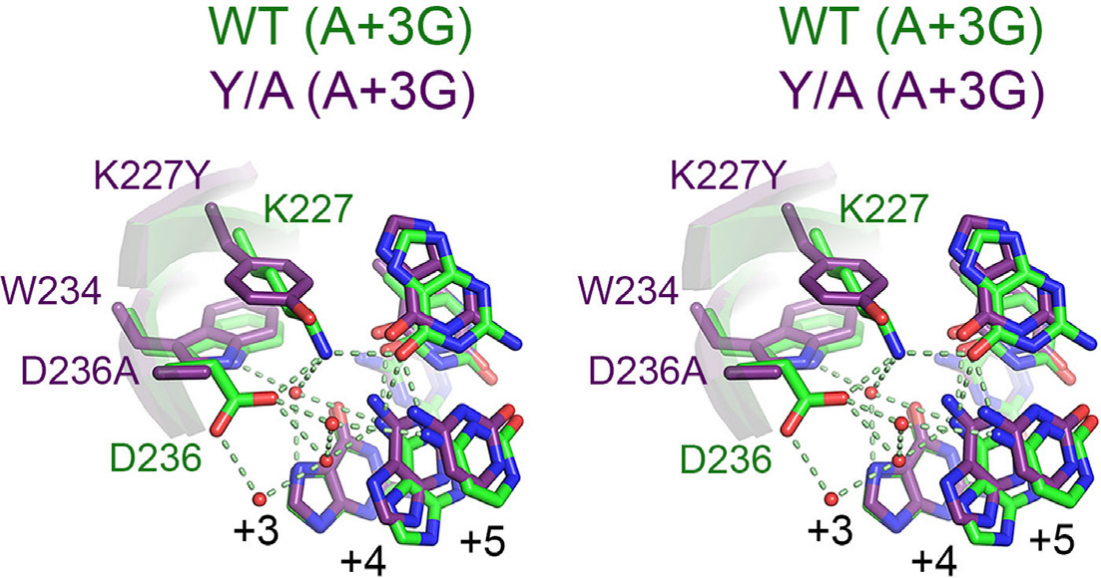
Significant changes in the network of water-mediated contacts between the wild-type and K227Y/D236A proteins and A+3G mutant substrates. Shown are stereo images with the wild-type protein and substrate colored green and the K227Y/D236A variant and substrate colored purple. Polar contacts are indicated by dashed lines.

**Figure 9.**
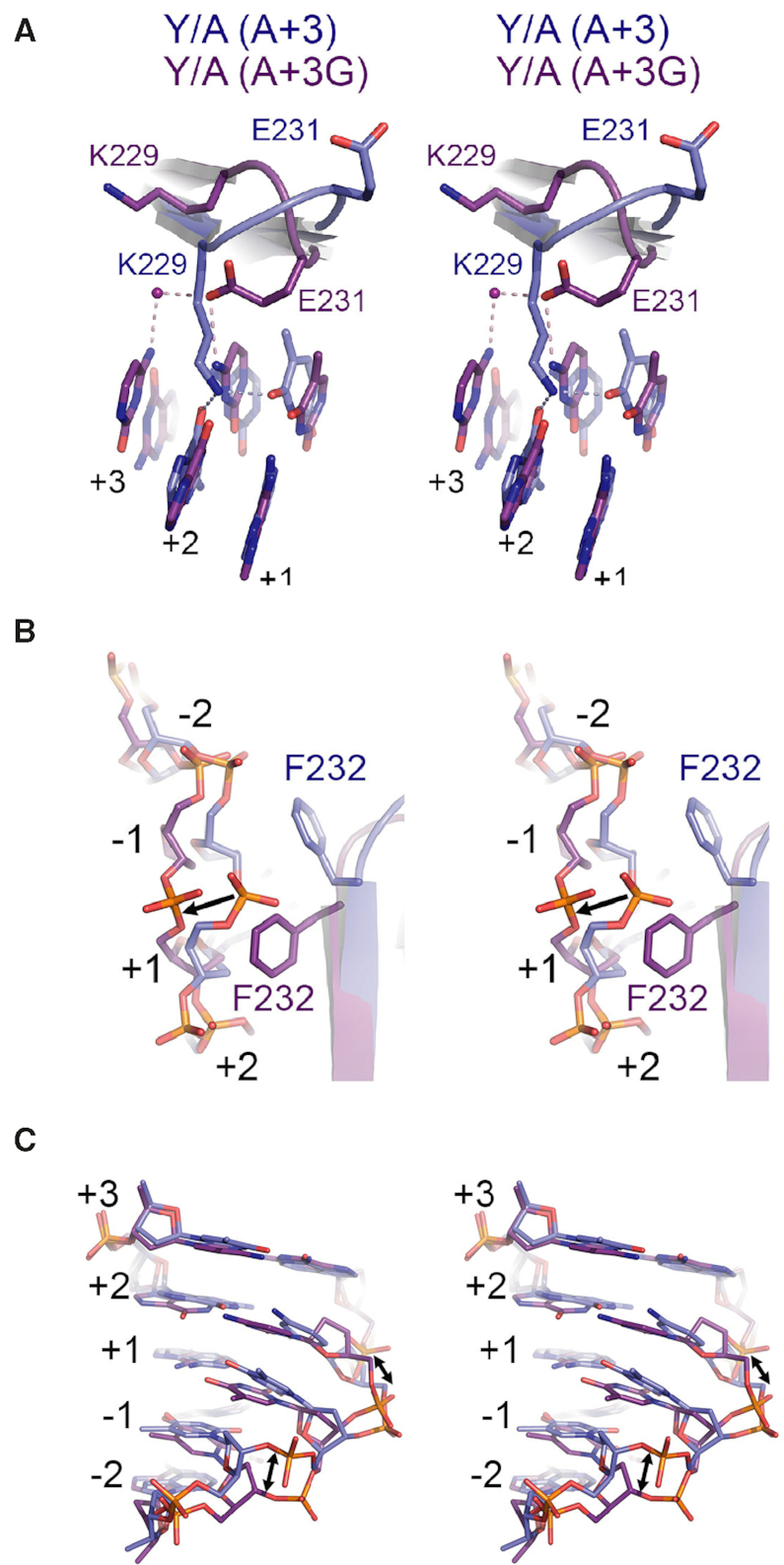
Changes in the hairpin loop and impacts on DNA structure in the K227A/D236A mutant. (**A**) Role swapping of the K229 and E231 residues on the A+3 cognate substrate (blue) and the A+3G substrate (purple). Polar contacts are shown as dashed lines and water molecules as spheres. (**B**) Rotation of F232 toward the phosphate backbone induces a shift in the A+G3 substrate. (**C**) Shifts in the phosphate backbone and bases in the A+3G substrate (purple) relative to the wild-type substrate (blue).

In co-crystals with the D227Y/D236A variant, the network of water mediated contacts between K227, W234 and D236 is significantly altered (Figure 8) such that antiparallel β-strands 7/8 are no longer stably anchored within the major groove. In turn, these strands and the hairpin loop joining them gain mobility, permitting substantial reorganization to accommodate the DNA structure in A+3G substrate. Of particular note is role swapping observed between residues K229 and E231 when the K227Y/D236A variant is bound to different substrates. When bound to A+3 substrate, residue K229 of the K227Y/D236A variant forms a direct contact with G+2 on the B DNA strand while residue E231 is entirely solvent exposed (Figure 9A). In contrast, when bound to A+3G substrate, K229 becomes solvent exposed while E231 forms a new direct contact with C+2 on the A DNA strand and a water-mediated contact with C+3 on the B DNA strand (Figure 9A). These changes are further accompanied by repositioning of F232 in the hairpin loop (Figure 9B), which rotates 120° and translates ∼1.5Å toward the +1 position causing a backbone translation of 3.5Å and local base distortions at +1, −1 and −2 nt (Figure 9C). Importantly, such movements leading to role swapping between K229 and E231 are not observed for the wild-type protein. Role swapping changes the pattern of hydrogen bond donors available for base contacts, and provides a rationale for the observed switch in C to T nucleotide preference of the K227Y/D236A variant at the +2 position.

## DISCUSSION

In this study, we used MI theory to predict covarying amino acid–DNA residues to gain insight into meganuclease specificity. We previously used MI theory to detect interchain covariation (that is, residue–residue covariation) in meganucleases, identifying a network of residues that was experimentally shown to regulate cleavage activity and specificity (37,39). Taken in context with the current data, our studies reaffirm findings that meganuclease DNA interactions comprise of direct and indirect readouts of DNA sequence and structure, and highlight how structural reorganization of an inter-hairpin loop can dramatically change substrate specificity. That similar reorganizations have been observed in other engineered meganucleases lends support to our data (30), while at the same time suggesting that extensive substrate profiling of engineered variants may reveal changes in substrate specificity that were not rationally incorporated into those meganuclease scaffolds.

One limitation of our study is the number of meganucleases that have characterized target sites. Because mis-alignments can increase the number of false positive predictions (32), it was critical to use characterized meganuclease target sites rather than bioinformatically predicted target sites. Doing so allowed us to accurately align the DNA sequences using the mapped top- and bottom-strand cleavage sites or orientation of the protein on substrate. Increasing the number of meganuclease-target site sequences could potentially reveal amino acid–DNA covariation with MIp scores that are below the level of significance with our current data set. It is important to note that the MI methodology we used provides a phylogenetically corrected score of residue–DNA interactions across an entire protein family (34) that must be interpreted within the context of individual family members. In I-OnuI, position K227 contacts the +5 position of substrate (17), whereas equivalent residues in other characterized meganucleases make direct contacts to the +3 base (R234 in I-LtrI and H222 in I-GzeII, for example) (17,19). Nonetheless, I-OnuI K227 is involved in a network of water-mediated contacts to adjacent residues (W234 and D236), perturbation of which results in the observed structural reorganization, shift of the neighboring DNA backbone and change in substrate preference for the K227Y/D236A variant relative to the wild-type enzyme.

The simplest interpretation of covariation analysis is that the identified residue pairs are in direct contact such that variation in one residue can be compensated by substitution in the second position to maintain local structure. Directed evolution experiments that identified substitutions in positions D236 and K227 that rescued cleavage on non-cognate DNA substrates support this interpretation. However, the largest structural compensations we observed involved residue (E231 and F232) and DNA (+2 and +1) positions that are outside of contact range of K227. In some cases, covariation analyses can identify putative functional dependency between non-contacting positions (5). In our case, covariation between K227, E231 and F232 was not detected for the simple reason that E231 and F232 lie in a loop region poorly conserved among meganucleases and were excluded from covariation predictions. From a structural perspective, it is not surprising that the most dramatic compensations were observed in loop regions with high flexibility and mobility.

Our data also reaffirm the generally accepted tenet that meganucleases are highly adaptable enzymes in the sense that the DNA-binding surfaces can adapt to different DNA substrates, either on an evolutionary timescale or in the laboratory. The residues we focused on here have been targeted by a number of directed-evolution studies to create variants with altered binding specificity at the +3, +4 and +5 positions of substrate (for example, (22–23,51–52)). Crystallographic studies of some of these engineered enzymes have noted similar changes to those we observed—restructuring of an inter hairpin loop, so-called role swapping where residues exchange DNA recognition for structural roles (or *vice versa*) in response to different DNA substrates, and distortion of the DNA backbone (30). An under-appreciated consequence of meganuclease adaptability is the potential to indirectly modulate cleavage preference at the central 4 bases. This modulation effectively expands the sequence space accessible to meganucleases for cleavage that in a biological context would enhance opportunities for transposition and invasion. It may be possible to expand meganuclease substrate range by layering on substitutions in active site residues that enhance activity on suboptimal substrates (39).

We suggest that some previously engineered meganucleases may have cleavage profiles that are unintended consequences of the re-design process, particularly those that were engineered to accommodate nucleotide substitutions in positions close to the central 4 bases. Engineering studies typically screen for activity on the desired target site versus native site by interrogating effects of single nucleotide substitutions, or by assessing cleavage at predicted off-target sites (for example, (53,54)). Many studies do not profile cleavage specificity on substrates with multiple nucleotide substitutions at adjacent positions, as we did here. Our data show that the readout of bases perturbed by re-engineering meganuclease–DNA contacts extends beyond the single targeted nucleotide position. From one perspective, the effects we observed would have the benefit of expanding the central 4 cleavage profile, which has previously been recalcitrant to rational engineering due to lack of direct base contacts. Conversely, the expanded substrate range could increase the number of off-target sites. Changes in cleavage preference would be difficult to predict from changes to meganuclease sequence alone. Comprehensive scans of substrate preference could be included in re-design workflows to better predict and minimize cleavage at off-target sites during therapeutic applications of meganucleases.

## DATA AVAILABILITY

Atomic coordinates and structure factors for I-OnuI structures have been deposited in the wwPDB under the following accession codes: I-OnuI wild-type, A+3G substrate: 6BDA; I-OnuI K227Y/D236A, A+3 substrate: 6BD0; I-OnuI K227Y/D236A, A+3G substrate, 6BDB.

## Supplementary Material

gkz866_Supplemental_Files

## Notes

Present address: Marc Laforet, Swift Medical, Toronto, ON M5H 3W4, Canada.

Present address: Thomas A. McMurrough, Specific Biologics, Toronto, ON M5G 0B7, Canada.
