# Supplementary material for: Modifying a covarying protein–DNA interaction changes substrate preference of a site-specific endonuclease
Source: Nucleic Acids Res. 2019 Oct 11;47(20):10830–41. doi: 10.1093/nar/gkz866 (PMC6847045; doi:10.1093/nar/gkz866)
Supplement: gkz866_Supplemental_Files [file gkz866_supplemental_files.zip › Laforet_supplementary_information_v2.pdf]

SUPPLEMENTARY INFORMATION: MODIFYING A COVARYING PROTEIN-DNA INTERACTION CHANGES SUBSTRATE PREFERENCE OF A SITE-SPECIFIC ENDONUCLEASE

MARC LAFORET, THOMAS A. MCMURROUGH, MICHAEL VU, CHRISTOPHER M. BROWN, KUN ZHANG,MURRAY S. JUNOP, GREGORY B. GLOOR, AND DAVID R. EDGELL

Table of contents:

(1) Supplementary Files
   (a) File S1 - Multiple sequence alignment of concatenated LAGLIDADG endonucleases and DNA target sites (provided as separate file).
(2) Supplementary Tables
   (a) Table S1 - Mutual information analysis (provided as separate file). Columns c and d are residue positions within the alignment used for analysis, and columns aa_c and aa_d are the residue identities. Column Zpx is the score used for plots in Figure 2 and for Table 1. Residue numbering is shifted relative to the I-OnuI crystal structure (3QQY) used as a reference (A21 is numbered A25). Numbering of amino acid positions stops at position 338 and the DNA substrates start at position 339.
   (b) Table S2 - k_{rel} rates for I-OnuI wild type, K227Y/D236A and D236E enzymes on all 256 substrates randomized at positions +2,+3,+4,+5 (provided as separate file). Data are reported as the mean of 5 biological replicates with the error reported as standard deviation from the mean.
   (c) Table S3 - Summary of crystallographic parameters (provided as separate file).
