# Supplementary material for: Modifying a covarying protein–DNA interaction changes substrate preference of a site-specific endonuclease
Source: Nucleic Acids Res. 2019 Oct 11;47(20):10830–41. doi: 10.1093/nar/gkz866 (PMC6847045; doi:10.1093/nar/gkz866)
Supplement: gkz866_Supplemental_Files [file gkz866_supplemental_files.zip › Table S3.pdf]

Table S3. Data collection and model refinement statistics

Parameter	Crystal 1	Crystal 2	Crystal 3
I-OnuI	Wild type	K227Y/D236A	K227Y/D236A
DNA substrate	A+3G	A+3	A+3G
PDB code	6BDA	6BD0	6BDB
Data Collection			
Space group	$P2_12_12_1$	$P2_12_12_1$	$P2_12_12_1$
Cell dimensions			
a, b, c (Å)	42.7, 69.0, 161.7	41.9, 66.7, 159.8	43.4, 67.2, 169.7
α, β, γ (°)	90, 90, 90	90, 90, 90	90, 90, 90
Resolution (Å)*	68.98-1.88(1.83-1.88)	66.7-1.45(1.46-1.45)	66.7-1.5 (1.52-1.5)
R_{merge} (%)*	6.5 (48.5)	4.6 (33.4)	5.6 (32.7)
I/ σ(I)*	7.9 (2.0)	8.3 (2.2)	10.1 (2.2)
Completeness (%)*	100 (100)	100 (100)	100 (100)
Redundancy*	5.7 (5.4)	7.6 (7.6)	5.9 (5.8)
Refinement			
Resolution (Å)	41.3 – 1.88	37.1 – 1.45	42.0 – 1.5
No. reflections	39721	80292	80414
R_{work}/R_{free} (%)	19.1/23.3	16.5/19.0	18.5/20.6
No. atoms	3864	4167	3983
B-factor (Å^2)	29.0	18.7	26.0
R.M.S deviations			
Bond lengths (Å)	0.006	0.019	0.012
Bond angles (°)	0.81	1.8	1.36

* Statistics for highest resolution shell are shown in parentheses.
